# Bibliometric analysis of postoperative deep vein thrombosis in total hip arthroplasty using CiteSpace

**DOI:** 10.3389/fsurg.2025.1585652

**Published:** 2025-05-22

**Authors:** Xi Ren, Changxu Han, Jiaxu Nie, Junwei Bai, Litian Zhang

**Affiliations:** ^1^ College of Nursing, Inner Mongolia Medical University, Hohhot, Inner Mongolia, China; ^2^ Sports Medicine Center, The Second Affiliated Hospital of Inner Mongolia Medical University, Hohhot, Inner Mongolia, China; ^3^ Quality Management Department, The Second Affiliated Hospital of Inner Mongolia Medical University, Hohhot, Inner Mongolia, China

**Keywords:** bibliometrics, deep vein thrombosis, aspirin, blood management, risk stratification

## Abstract

**Background:**

There was a lack of bibliometric analyses of postoperative deep vein thrombosis (DVT) after total hip arthroplasty (THA), and this study aimed to provide a comprehensive overview of the knowledge structure and research hotspots in this area through visual analyses.

**Method:**

The Web of science (WOS) core databases were searched for relevant studies built up to March 2024, and CiteSpace was then used to create a network diagram, analyze the authors, institutions, nations, journals, keywords, and references in this field generally, as well as to investigate hotspots and trends in research in this field.

**Results:**

There were 1,299 pertinent papers in all, and the number of publications in the topic was generally rising. The author with the highest number of publications is Parvizi, Javad, and the institution is Jefferson University, while the United States is the most influential and contributing country in the field, the top 5 high frequency keywords are venous thromboembolism, deep vein thrombosis, prevention, total hip arthroplasty, replacement; the keyword dabigatran etexilate has the highest burst intensity in burst detection, while aspirin, blood management, and risk stratification are emerging research trends.

**Conclusion:**

This study examines the literature on postoperative DVT following THA using CiteSpace, which offers useful data for possible cooperation between authors, countries, and research institutions. It also identifies hotspots and trends for future research, which will be a resource for scholars looking to delve deeper into the preventive measures for DVT following THA.

## 1 Introduction

Total hip arthroplasty (THA) is the typical surgical technique for hip fractures and ischemic necrosis of the femoral head (1, 2). THA surgery rates have increased by 7.02% on average per year. It is estimated that by 2030, there will be 572,000 cases of THA (3, 4). Deep vein thrombosis (DVT) is a common complication following THA. In patients who do not receive thromboprophylaxis, the incidence can be as high as 42%–57%, leading to increased readmission rates, mortality, and healthcare costs (5–7). Since 1950, researchers have been examining postoperative orthopaedic DVT; more recently, the prevention of postoperative DVT has taken on a more personalized and in-depth nature and is still a topic of substantial investigation (8).

Bibliometrics is a scientific method of analysing literature, either quantitatively or qualitatively, to understand the distribution of contours and trends in a particular field of research (9). CiteSpace is a widely used bibliometrics analytical tool that is based on the Java language for visual analysis. Through the co-occurrence, clustering and bursting analyses of different types of nodes, it demonstrates the research hotspots of a particular discipline or knowledge area in a given period of time in the form of a visual network graph and analyzes its potential development trend more thoroughly, intuitively, and scientifically (10, 11).

There is currently no bibliometric study specifically focused on DVT following THA, resulting in an unclear knowledge structure and emerging trends in this field. Therefore, this study aims to use CiteSpace software for a visual analysis of the included literature, with the goal of identifying research hotspots and trends in this area, providing new academic perspectives and references for the prevention and treatment of postoperative DVT.

## 2 Method

### 2.1 Literature sources and search strategy

This study chose the Web of Science (WOS) Core Collection database for literature retrieval. The WOS journal selection criteria are stringent, covering high-quality, high-impact peer-reviewed journals, and providing complete citation data, which helps reduce the interference of low-quality literature (12). The retrieval time span was from the establishment of the database until March 2024. “TS = total hip replacement OR TS = total hip arthroplasty) AND (TS = Deep Vein Thrombosis OR TS = Postoperative deep vein thrombosis OR TS = Venous thromboembolism)” is the search formula used in this subject-based search. This study focused on DVT following THA; clinical trials and reviews were the forms of literature that were included; duplicates, material irrelevant to the issue term, and literature lacking information were eliminated.

### 2.2 Inclusion and exclusion criteria

Inclusion Criteria: ① Study subjects: Patients undergoing THA; ② Research topic: DVT; ③ Study type: Clinical studies (randomized controlled trials, cohort studies) or reviews. Exclusion Criteria: ① Non-English language literature; ② Animal studies, case reports, reviews; ③ Incomplete data or inaccessible full-text articles; ④ Duplicate publications. The literature screening was independently conducted by two researchers, with any discrepancies resolved through consultation with a third researcher.

### 2.3 Literature analysis methodology

**Figure 1:**
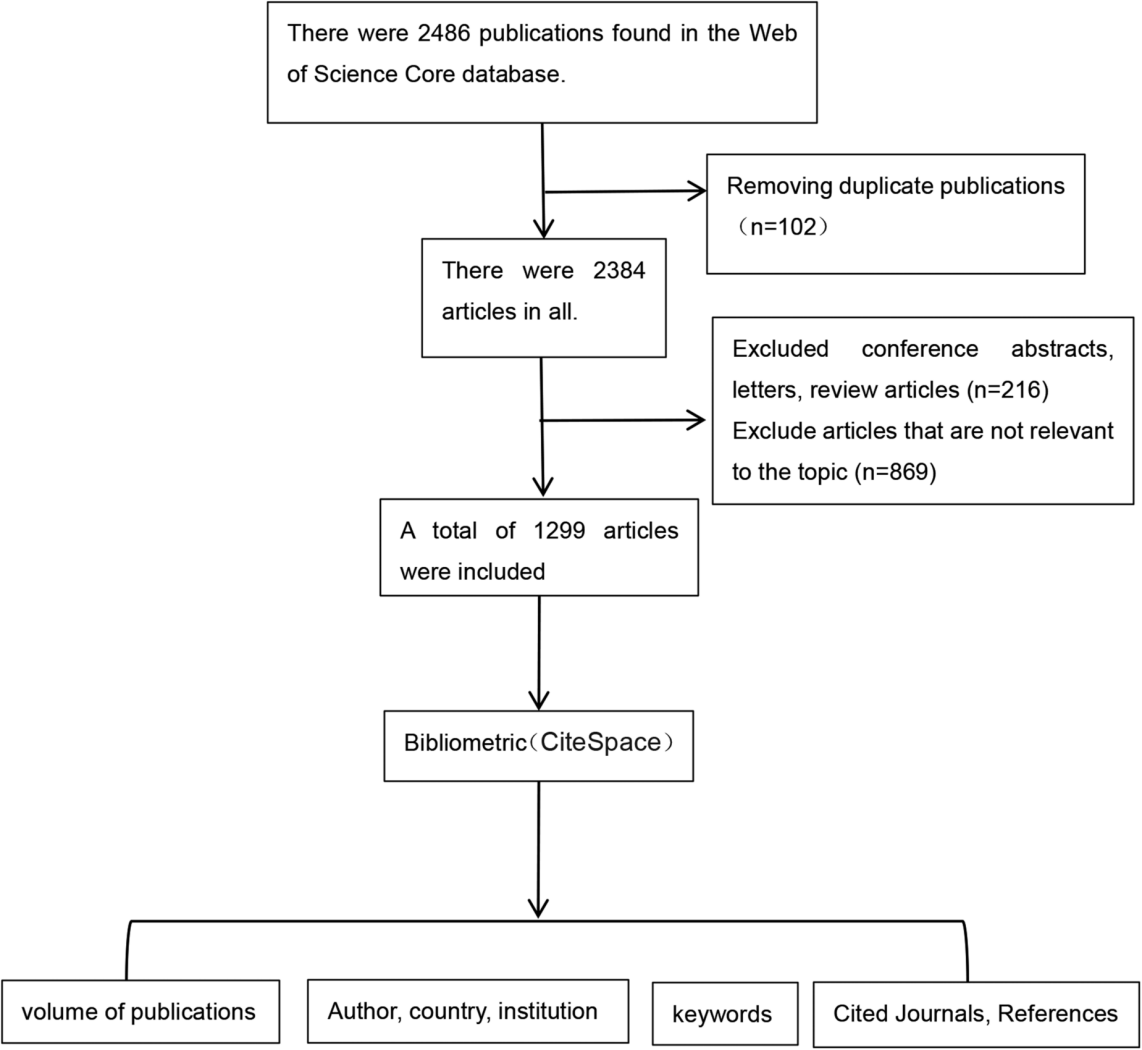
Bibliometric analysis flowchart.

For analysis and visualization, the included literature was imported into CiteSpace 6.3.R1 in plain text format. Figure 1 illustrates the analysis procedure. Configuring the analysis parameters: time span 2007–2024. The node kinds are “Author”, “Institution”, “Country”, “Reference”, “Cited Journal” and “Keywords”. The time slice was set to 1. The threshold criterion Top *N* is set at 50 and the pruning method is “pathfinder” “pruning the merged networks”. This study followed the *Preliminary guideline for reporting bibliometric reviews of the biomedical literature (BIBLIO)* statement guidelines (13).

## 3 Results

### 3.1 Annual number of articles and trends

**Figure 2:**
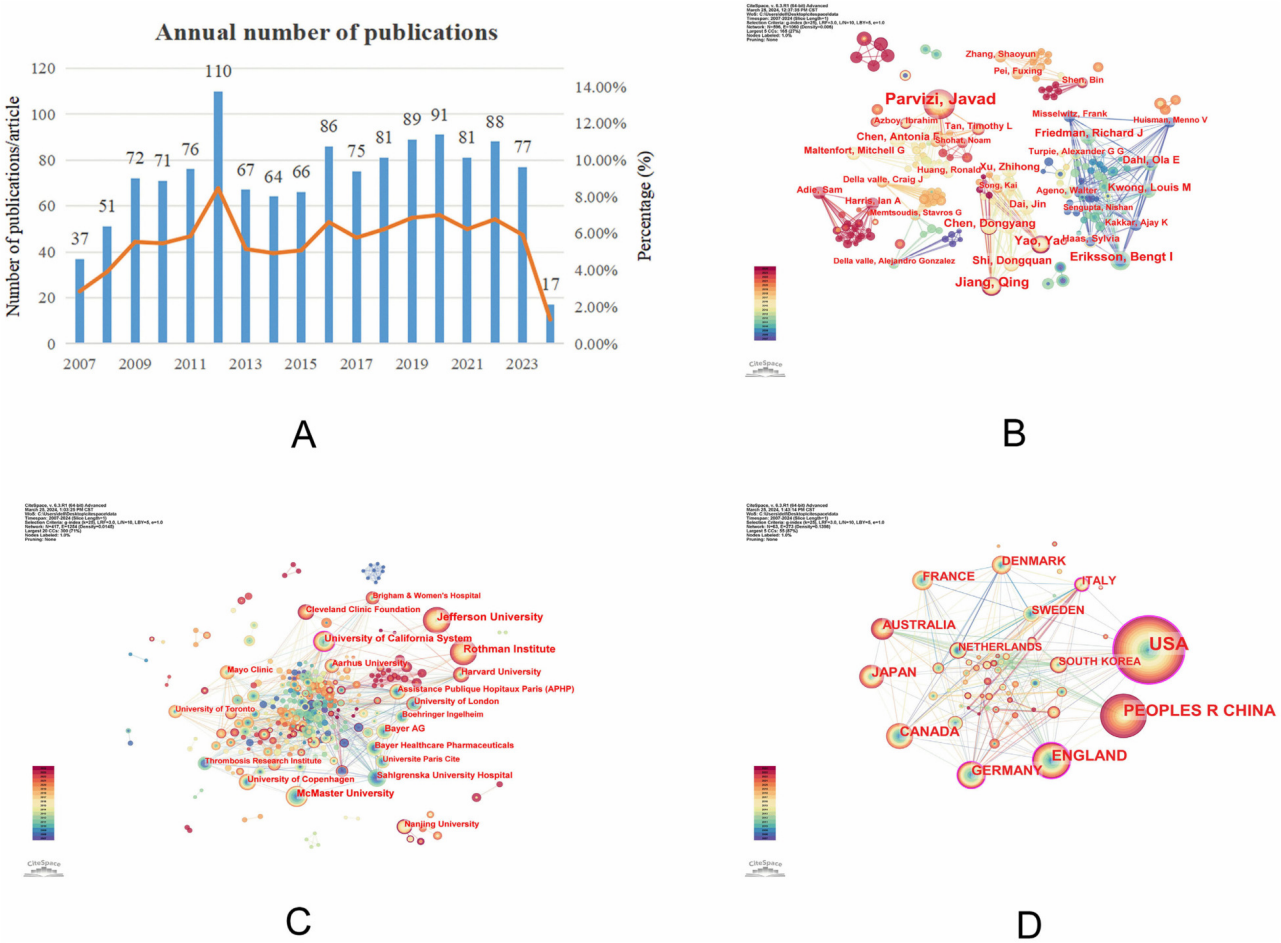
**(A)** Trend graph of growth in the number of publications; **(B)** graph of co-occurrence analysis of authors of postoperative deep vein thrombosis after total hip arthroplasty; **(C)** institutional co-occurrence analysis graph of postoperative deep vein thrombosis after total hip replacement; **(D)** country co-occurrence analysis chart for postoperative deep vein thrombosis after total hip arthroplasty.

A total of 1,299 papers pertaining to DVT following THA were eventually included after screening in accordance with the inclusion criteria. In this field, the number of publications began to rise in 2007 and peaked in 2011, after which there was an overall fluctuating upward trend in the number of articles published. From 2024 to March 2024, there were 17 pertinent studies published in this field; this suggests that the field will continue to see relatively stable research intensity in the long run (Figure 2A).

### 3.2 Co-operation analysis

#### 3.2.1 Author analysis

To analyze the core authors and the degree of collaboration among them in this field, a collaboration network analysis was conducted. The network consists of 596 nodes and 1,060 connections, with a network density of 0.006. The most influential and prolific author is Parvizi, Javad, with 46 published articles. He is followed by Jiang, Qing (20 articles) and Eriksson, Bengt I (16 articles). Parvizi, Javad has been an active author from 2015 to 2024. Adie, Sam and Harris, Ian A are recent additions to the field (Figure 2B, Table 1).

**Table 1:** Top 10 co-authors, institutions, and countries in the field of deep vein thrombosis research after total hip arthroplasty.

No.	Author	Volume of publications	Centrality	Institutional	Volume of publications	Centrality	Country	Volume of publications	Centrality
1	Parvizi, Javad	46	0.01	Jefferson University	66	0.03	USA	485	0.45
2	Jiang, Qing	20	0.00	Rothman Institute	61	0.04	Peoples R China	220	0.03
3	Eriksson, Bengt I	16	0.01	University of California System	40	0.16	England	139	0.22
4	Yao, Yao	16	0.00	McMaster University	40	0.10	Germany	89	0.12
5	Chen, Dongyang	12	0.00	Sichuan University	29	0.00	Canada	86	0.02
6	Friedman, Richard J	11	0.00	Harvard University	28	0.09	Japan	75	0.00
7	Shi, Dongquan	11	0.00	Bayer AG	27	0.03	Australia	58	0.05
8	Dahl, Ola E	10	0.00	Aarhus University	26	0.06	France	52	0.04
9	Kwong, Louis M	10	0.00	Sahlgrenska University Hospital	25	0.05	Denmark	52	0.02
10	Chen, Antonia F	10	0.00	University of Copenhagen	23	0.06	Italy	41	0.15

Despite the clear team relationships visible in the collaboration network graph, both the network density and author centrality are low. This indicates that the level of collaboration between authors in this field is still insufficient.

#### 3.2.2 Institutional analysis

A representation of the WOS literature using institutions as nodes revealed 417 nodes, 1,254 connection points, and 0.0145 connection density. Jefferson University (66), Rothman Institute (61), and University of California (40) are the top three institutions in terms of publications (Figure 2C, Table 1).

Among the top ten institutions, only the University of California has a centrality greater than 0.1. This suggests that the University of California has strong collaborative relationships with other institutions.

#### 3.2.3 Country analysis

The analysis of publishing countries revealed the following: The USA has the highest number of publications, with 485 papers; it's also ranks first in centrality. This suggests that the USA is the most influential in this field of academic research and has strong international collaboration with other countries (Figure 2D, Table 1).

### 3.3 Keyword analysis

#### 3.3.1 Keyword co-occurrence and cluster analysis

**Figure 3:**
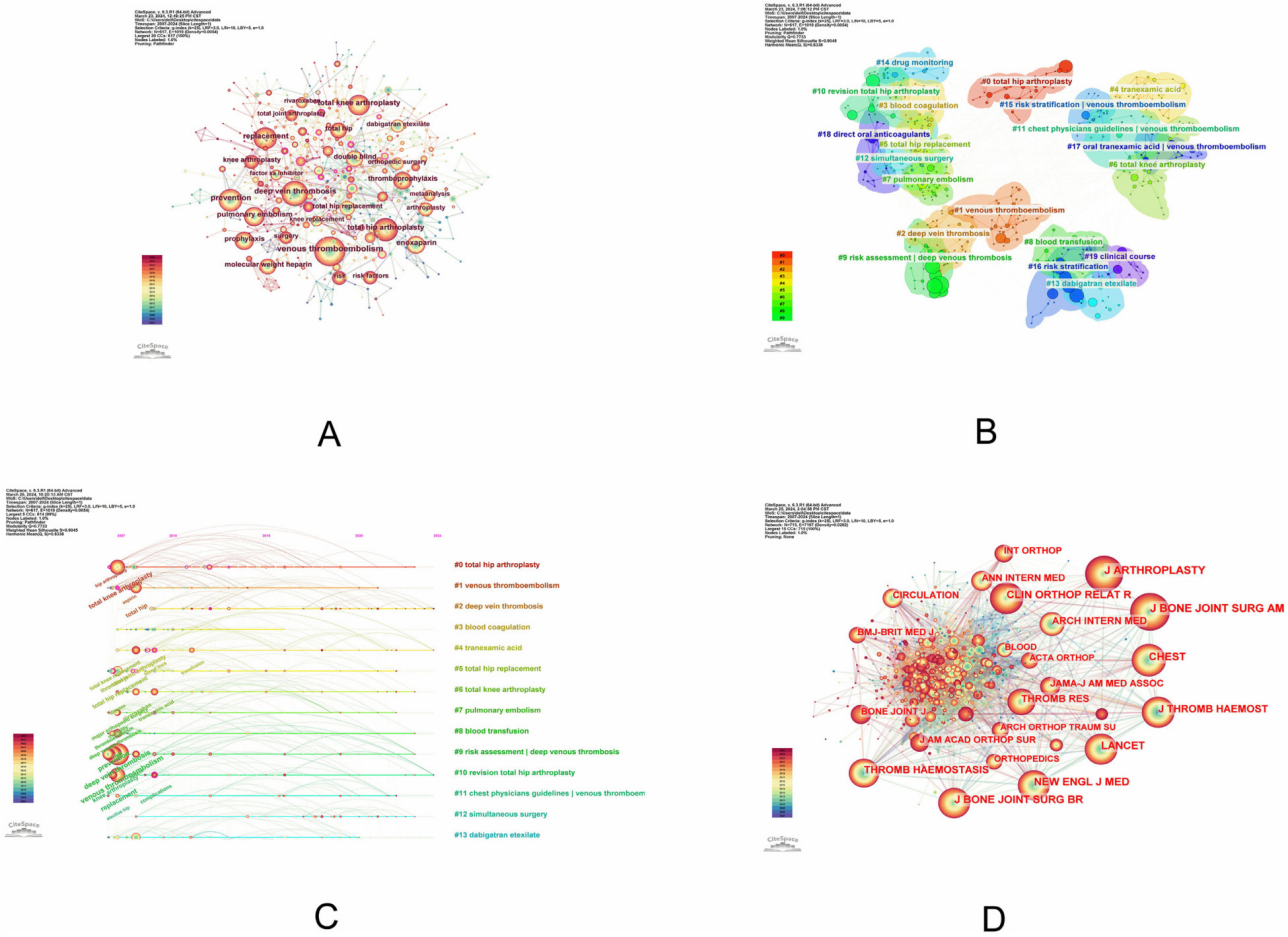
**(A)** Keyword co-occurrence analysis graph for postoperative deep vein thrombosis after total hip replacement; **(B)** keyword cluster analysis graph for postoperative deep vein thrombosis after total hip replacement; **(C)** keyword timeline graph for postoperative deep vein thrombosis after total hip replacement; **(D)** keyword co-occurrence analysis graph for cited journals for postoperative deep vein thrombosis after total hip replacement.

A keyword is a synopsis of a study area's subject matter that might reveal research trends, hotspots, and present state. Figure 3A displays the findings of the co-occurrence of keywords. There were 617 nodes, 1,019 connections E, and 0.0054 network density. Venous thromboembolism, deep vein thrombosis, prophylaxis, total hip arthroplasty, and replacement were the top 5 high-frequency keywords, based on keyword occurrence frequency (Table 2). When it comes to keyword clustering, *Q* > 0.3 is regarded as significant, *S* > 0.5 suggests that the grouping is acceptable, and *S* ≥ 0.7 indicates that the results are convincing. In this study, the keyword clustering results (*Q* = 0.7733, *S* = 0.9045) are both significant and persuasive (14). The keywords were aggregated into 20 categories, indicating the primary current concerns in the field. The top 5 items were #0 total hip arthroplasty, #1 venous thromboembolism, #2 deep vein thrombosis, #3 blood coagulation, and #4 tranexamic acid (Figure 3B).

**Table 2:** Top 20 high-frequency keywords in the research field of postoperative deep vein thrombosis after total hip arthroplasty.

No.	Keyword	Year of initial appearance	Frequency	Centrality
1	Venous thromboembolism	2007	582	0.01
2	Deep vein thrombosis	2007	430	0.01
3	Prevention	2007	373	0.00
4	Total hip arthroplasty	2007	350	0.01
5	Replacement	2007	317	0.02
6	Total knee arthroplasty	2007	307	0.00
7	Pulmonary embolism	2007	260	0.03
8	Enoxaparin	2007	251	0.01
9	Thromboprophylaxis	2007	237	0.00
10	Prophylaxis	2007	223	0.00
11	Double blind	2007	205	0.01
12	Molecular weight heparin	2007	205	0.00
13	Total hip	2008	179	0.01
14	Knee arthroplasty	2007	174	0.03
15	Total hip replacement	2007	165	0.00
16	Arthroplasty	2007	153	0.05
17	Surgery	2007	149	0.05
18	Dabigatran etexilate	2008	145	0.02
19	Risk	2007	143	0.05
20	Risk factors	2007	141	0.01

#### 3.3.2 Analysis of keyword timeline graphs

The dynamic evolution route of the research hotspots represented by the keywords may be seen on the timeline graph. In a particular research subject, it can also reveal the peaks and valleys of term popularity as well as the temporal features of keyword clustering (15). The analysis of the keyword timeline graph of the WOS database shows that global researchers have studied DVT after THA throughout the course of the journey, and the curves in the graph indicate the degree of connectivity between the keyword nodes. Of them, the field was most affected by #9 risk assessment | deep venous thrombosis in the beginning. Deep vein thrombosis #2, tranexamic acid #4, and revision total hip arthroplasty #10 clustering in 2024 still show up and continue to propel this study area forward (Figure 3C).

#### 3.3.3 Keyword burst analysis

**Figure 4:**
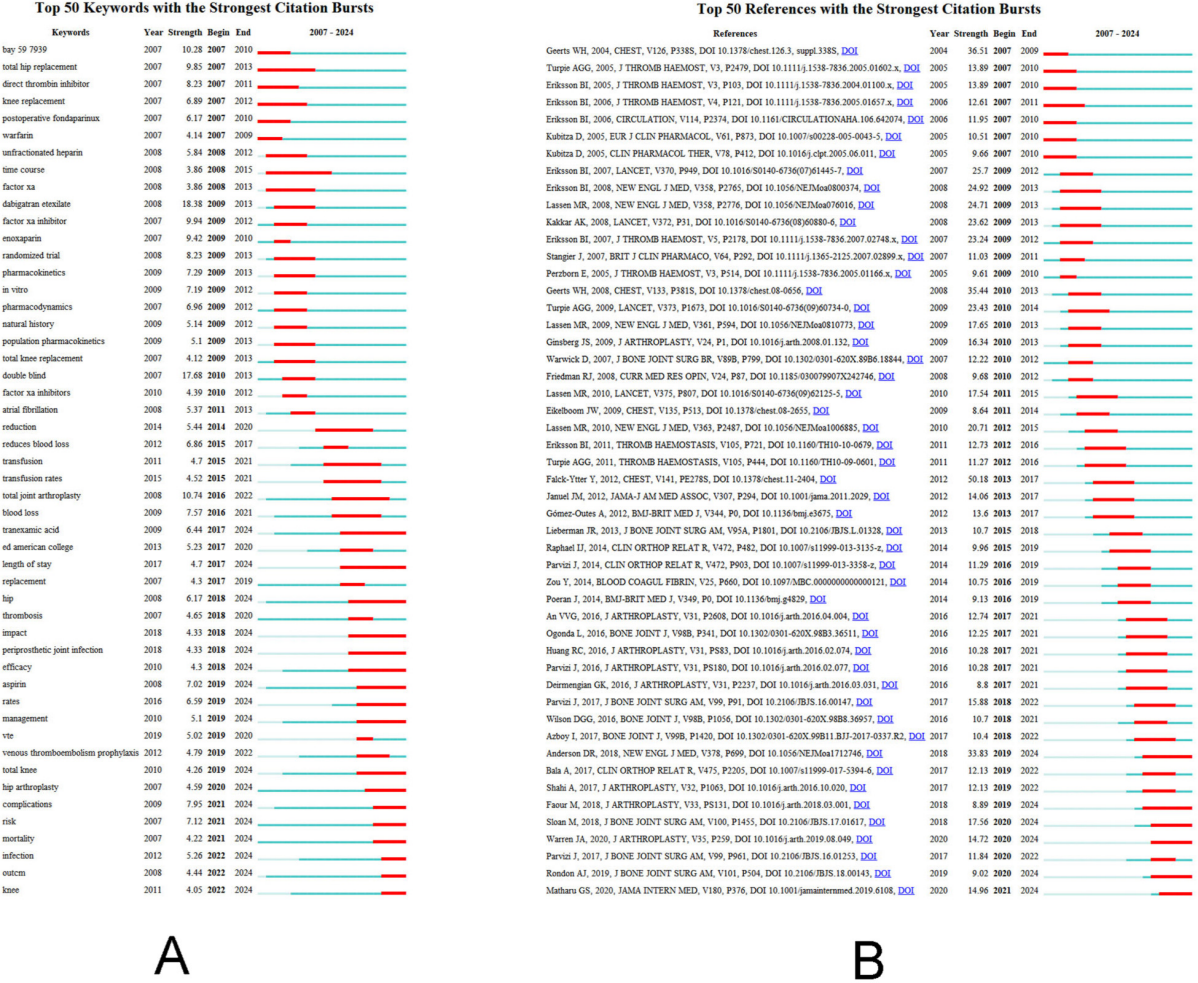
**(A)** Keyword burst analysis of postoperative deep vein thrombosis after total hip arthroplasty; **(B)** cited literature burst analysis of postoperative deep vein thrombosis after total hip arthroplasty.

Keyword burst identification identifies hotspots and innovative study directions by displaying the length and intensity of a keyword burst in the field over a predetermined amount of time (16). Figure 4A demonstrates that the primary focus during the early years of the field (2010) was on “anticoagulant drug”, “pharmacokinetics”, “total hip replacement” and “direct thrombin inhibitor”. Key words like “blood loss” and “transfusion” started to appear after 2010, and the decrease in blood loss or blood transfusion rate became a focus of postoperative DVT research following THA; after 2018, aspirin, an alternative medication, postoperative DVT “complications” and “risk factor” gained a lot of attention. Furthermore, the burst map reveals that “tranexamic acid” and “length of stay” has the longest outbreak duration of 2017–2024, while “dabigatran etexilate” has the greatest burst, with a burst intensity of up to 18.38. Research on “Aspirin”, “rates”, “management”, “total knee”, “hip arthroplasty”, “complications”, “risk”, “mortality”, “infection”, “outcm” and “knee” is expected to pick up steam in 2019.

### 3.4 Analysis of journal co-citation and cited literature bursts

The top 10 journals by citation count are displayed in Figure 3D, Table 3, with Lancet having the highest impact factor (IF = 168.9). With up to 908 citations, publication of Journal of Arthroplasty was the publication with the highest frequency of cited references. This indicates that research articles published in the journal about DVT following total hip arthroplasty are very influential in the field and frequently mentioned.

**Table 3:** Top 10 cited journals in the field of postoperative deep vein thrombosis research in total hip arthroplasty.

No.	Journal	Citation frequency	Impact factor	JCR partition
1	Journal of Arthroplasty	908	3.5	Q1
2	Journal of bone and joint surgery American volume	862	5.3	Q1
3	Chest	755	10.1	Q1
4	Lancet	700	168.9	Q1
5	Journal of bone and joint surgery British volume	682	-	-
6	Clinical Orthopaedics And Related Research	679	4.3	Q1
7	New England Journal of Medicine	675	158.5	Q1
8	Journal of Thrombosis And Haemostasis	589	10.4	Q1
9	Thrombosis And Haemostasis	564	6.7	Q1
10	Thrombosis Research	491	7.5	Q1

The top 50 references with the biggest citation bursts are shown in Figure 4B. The 2012 publication “Falck-Ytter Y, 2012, CHEST, V141, PE278S, DOI 10.1378/chest.11-2404” had the strongest citation burst (17). The burst's intensity was 50.18. This article, which has an impact factor of 10.1 and was published in the journal Chest, focuses on describing the antithrombotic pharmacological and nonpharmacological approaches to orthopaedic surgery (hip arthroplasty), offering the best preventive methods for postoperative DVT, and indicating the direction that DVT prevention should take going forward. A clinical trial with an outbreak intensity of 33.83 that was published in the New England Journal of Medicine in 2018 is one of the higher citation outbreak intensities in recent years. In patients who underwent total hip or total knee arthroplasty, postoperative prophylaxis with aspirin did not significantly differ from rivaroxaban in preventing postoperative venous thrombosis, according to this multicenter, double-blind, randomised controlled trial (18). Aspirin is also reasonably priced and may be a valuable long-term thrombosis prophylaxis option following total hip arthroplasty.

## 4 Discussion

### 4.1 Basic information

This study summarizes the cutting-edge trends and research hotspots in the field of DVT following THA and visualizes the research profile through CiteSpace.

Since 2007, there has been a general trend of activity in the sector with an increase in publications, most likely because of an aging population and an increase in patients undergoing THA. The most prolific and continuously active author is Parvizi, Javad, who has four of the top 50 references in terms of citation bursts, with burst intensities of 11.29, 10.28, 15.88, and 11.84, respectively, indicating a prolonged impact in the area. The institutional collaboration network map reveals that the majority of the institutions working in this field are hospitals and universities, with a small number of pharmaceutical companies. Given the significance of pharmacological prevention of thrombosis, it is strongly advised that all types of institutions engage in extensive collaboration and communication. Research in this field is conducted in countries all over the world. According to an examination of country network collaboration, of which the United States is the most prominent contributor and cooperates most closely with other countries. This is most likely due to the high prevalence of THA that existed in the US between 1990 and 2002, and its prevalence has continued to rise (19), which has contributed to the early emergence of scholarly research and the building of academic competence in this field. Furthermore, the United States has significantly more scientific and technological advancements as well as economic strength than other nations. Government and researcher support for medical research has also led to a higher number of scientific research findings in this area (20). In the future, it will be necessary to foster the development of an international system of academic network cooperation in this field and to raise the level of cooperation across countries and regions. In addition, referenced journals have an impact factor as high as 168.9. The distribution of journals in the area is shown by the cited journal analysis, which helps to publish academic findings and gives researchers pertinent information about which journals to submit manuscripts to.

### 4.2 Research hot spots and future trends

Keywords reflect the core of research, and emergent analyses help to understand the cutting-edge of research (9). In this study, keywords were analysed using CiteSpace to summarise representative emerging research trends in DVT after THA.

#### 4.2.1 Alternative thromboprophylaxis: aspirin

Anticoagulant therapy plays a critical role in the prevention of DVT following THA. These drugs achieve the goal of reducing thrombosis by interfering with the key links of the coagulation cascade reaction, inhibiting the generation or activity of thrombin, and thereby blocking the conversion of fibrinogen to fibrin (21). Commonly used anticoagulants include injectable agents (low molecular heparin, fondaparinux), warfarin, and novel oral anticoagulants (dabigatran, rivaroxaban) (22). Each class of drugs exhibits distinct mechanisms of action and clinical characteristics, enabling tailored treatment for diverse patient populations. Low molecular heparin and fondaparinux can effectively prevent thrombosis via subcutaneous administration without the need for continuous monitoring of anticoagulant efficacy, thus facilitating outpatient or home-based DVT management (23). However, these agents should be used cautiously in patients at higher risk of bleeding (24). Warfarin provides the advantage of oral administration, eliminating the need for mandatory subcutaneous injections (25). However, this anticoagulant has a narrow therapeutic window, requiring frequent coagulation monitoring. Additionally, its interactions with medications and certain foods may increase bleeding risks (26). Novel oral anticoagulants demonstrate comparable or superior efficacy to traditional anticoagulants in the prevention of DVT post-THA, with the added advantage of minimizing bleeding risks to the greatest extent possible (27, 28).

Although anticoagulant drugs have demonstrated unique advantages in preventing DVT following THA, aspirin has emerged as an alternative drug for DVT prevention due to its cost-effectiveness, lack of requirement for subcutaneous injection, and absence of the need for regular blood monitoring (22). Aspirin, a non-steroidal anti-inflammatory drug with anti-platelet aggregation properties, has been proven effective in secondary cardiovascular disease prevention, improving patient outcomes (29). Relevant guidelines list aspirin as an alternative preventive option for venous thromboembolism after THA (17, 30, 31). From 2011 to 2019, the use of aspirin post-THA gradually increased (32). Burst detection analysis of key terms indicates that aspirin's highest burst intensity occurred between 2019 and 2024, reaching a value of 7.02, marking it as an emerging research area. Clinical studies have shown that aspirin exhibits comparable efficacy to rivaroxaban in DVT prevention without increasing the incidence of bleeding events compared to other anticoagulants (22). However, a non-inferiority randomized controlled trial found no statistically significant advantage of aspirin over enoxaparin in DVT prevention after THA (33). Additionally, there remains a lack of clear guidance regarding the optimal dosage and duration of aspirin for DVT prevention. Clinical judgments made by physicians regarding a patient's dosage of thrombosis prophylaxis are influenced by the patient's thrombosis features, tolerance, and complications as well as their own experience and preferences (34). A related study discovered that the best cost-effective way to reduce the incidence of pulmonary embolism and hemorrhage following THA was to administer regular dose, regular duration low molecular heparin for 10 days, followed by low dose, extended duration aspirin for 28 days (35). Nevertheless, it is critical to note that gastrointestinal reactions represent the most common adverse effects of aspirin. Even at lower doses, aspirin can elevate the risk of upper gastrointestinal bleeding, which, if untreated promptly, may endanger patients' lives (36, 37).

#### 4.2.2 Perioperative blood management

One of the most frequent side effects of THA is anemia. Research has shown that postoperative anemia in THA patients is linked to decreased preoperative hemoglobin levels, lengthy surgical procedures, and higher intraoperative hemorrhage (38). Improved perioperative blood management, which frequently uses tourniquets, blood transfusions, hemostatic medications, and tranexamic acid, can successfully minimize THA-associated anemia (39). Nonetheless, a substantial amount of data indicates that anemia patients receiving red blood cell transfusions had a 1.39-fold higher risk of developing postoperative venous thrombosis, and that perioperative red blood cell transfusions may even encourage the development of postoperative DVT. The accumulation of physiologically reactive chemicals resulting from particular blood storage may be the cause, together with the pro-hypercoagulant impact of erythrocyte infusion exacerbating the formation of physiological or pathological thrombi (40, 41). An essential component of the perioperative blood management of THA is tranexamic acid (TXA), an amino acid analogue with antifibrinolytic properties that lowers the rate of postoperative bleeding and transfusion and improves anemia in patients with THA but raises the risk of venous thromboembolism (42–44). Relevant research has revealed that intravenous and local TXA have comparable effects on hemostasis and do not raise the risk of venous thromboembolism. Additionally, local injections decrease plasma TXA concentrations and limit systemic absorption of TXA, which is more advantageous for patients who are more likely to experience thromboembolism (39). In addition, perioperative blood loss can be significantly decreased by combining two blood management strategies. It was discovered that in patients with THA, perioperative blood loss and blood transfusion can be successfully decreased by using hypotensive anesthesia approaches to control intraoperative bleeding during surgery and TXA to prevent fibrinolytic activation (45). When it comes to keyword analysis, TXA stands out more since it not only shows up as a distinct cluster but also lasts the longest in burst detection—an emerging subject that warrants further investigation. Studies on DVT following TXA and THA are currently scarce, and high-caliber research is required to thoroughly examine the ideal TXA dosage, the benefits of the combination, and any potential negative consequences.

#### 4.2.3 Risk stratification and machine learning

After THA, risk stratification is crucial for determining the best possible balance between bleeding and DVT as well as for determining the best individualized thromboprophylaxis plans for individuals at various risk levels (46). Regarding the optimal method for risk stratification, there is no agreement (47). The most popular technique for determining thrombosis following orthopaedic surgery is called caprini risk, which also assesses the patient's degree of DVT risk. However, it has drawbacks, including high prediction error, lack of dynamic warning, and time consumption (48, 49). Chen (50) suggested thromboelastography as a risk classification tool, however its projected viability was poor. In order to determine which patients with THA were at low and high risk and to prescribe different anticoagulants, Johnson (51) developed a risk stratification tool. For low-risk patients, aspirin use decreased the rate of bleeding events and did not increase the number of DVT events; but it additional external validation of its effectiveness is required.

Artificial intelligence (AI), of which machine learning is the primary subfield, has gained significant traction in academia as a result of the development of healthcare technology. Machine learning (ML) is an algorithm that is driven by data and is based on finding patterns and trends in data, analyzing patterns in data, and coming to conclusions and predictions (52). In contrast to conventional analysis techniques, machine learning can more accurately and efficiently discover non-linear correlations between variables by mining and exploring deeper and more complicated interactions between data (47). Machine learning has been shown to be more effective at predicting DVT following THA by repeated cross-validation of various models (53). Compared to Traditional Risk Assessment Tools, Machine Learning Models Offer Higher and More Accurate Predictability of DVT After THA. It was discovered that all machine learning models had an area under the curve (AUC) >0.88. A combination of extreme gradient boosting (XGBoost), random forest (RF), support vector machine (SVM), and logistic regression (LR) models had the highest predictive value for DVT with an AUC of up to 0.9206 (54). Nevertheless, postoperative DVT following THA and machine learning remain a relatively unexplored field, and the application of machine learning may miss certain important complicating factors. To enable individualized thromboprophylaxis for patients and enhance patient prognosis, it will be necessary to integrate complicated variables that have not yet been evaluated and to enhance machine learning-based prediction tools in the future.

### 4.3 Future research trend

This study provides a detailed visualization analysis of the knowledge structure and research trends in DVT after THA, offering researchers a deeper understanding of the field's development. In clinical practice, guideline-recommended alternatives such as aspirin can be used with personalized consideration. ML-based risk stratification models, integrated with electronic health records, may enable real-time patient data analysis, allowing clinicians to adjust anticoagulation strategies dynamically and reduce over-treatment risks. However, several unresolved issues remain. While both aspirin and anticoagulants have advantages and limitations in preventing DVT after THA, their efficacy and safety require further validation through large-scale, multicenter randomized controlled trials. Perioperative blood management, particularly the use of TXA to reduce blood loss, is a current research priority. Yet, optimal dosing, combination therapies, and safety profiles of TXA remain unclear (55), necessitating high-quality studies to assess its impact on DVT risk. Risk stratification and machine learning are emerging as promising tools for DVT prevention post-THA. Governments should support cross-institutional data-sharing platforms and establish privacy-protection laws to facilitate AI integration in clinical practice. However, the internal and external validity of risk stratification models remains unverified (56), and ML-based predictive models lack generalizability (57). Future research should prioritize multicenter validation to enhance their reliability.

### 4.4 Strengths and limitations

This study employs bibliometric methods to comprehensively analyze the knowledge structure and research hotspots of DVT following THA, providing valuable insights into the current state and future directions of research in this field. However, there are several limitations to this study. First, due to the limitations of the software used, only articles from the WOS core database were included in the analysis, which may exclude important findings from other databases, potentially leading to selection bias. Despite this, visualizing research trends in a specific field remains highly significant. Second, this study used CiteSpace for bibliometric analysis, which cannot assess the quality of individual articles. Third, the visualization software could not identify all authors of the included studies, and as a result, only the first authors were analyzed, preventing an evaluation of the contributions of other authors in this field.

## 5 Conclusion

In conclusion, based on CiteSpace, this study offers a visual review of pertinent research on DVT following THA. The field is seeing an increase in publications overall, but there is a lack of collaboration between research authors, institutions and countries, and future trends in research priorities are likely to revolve around “aspirin”, “blood management”, “risk stratification and machine learning”. However, there is still a need for large-sample, multi-centre randomised controlled trials around the hot trends in research, and in-depth research was done on the best way to administer thromboprophylaxis medications, and the machine learning model for predicting the risk of thrombosis was improved to serve as a guide for creating individualized thromboprophylaxis plans.

## Data Availability

Data from this study are available upon request from the corresponding author.
